# The role of vascular resistance on coronary perfusion: differences between healthy and anomalous coronary origin under rest and exercise conditions

**DOI:** 10.3389/fcvm.2026.1881521

**Published:** 2026-10-13

**Authors:** Seyyed Mahmoud Mousavi, Gianluca Zitti, Marco Pozzi, Maurizio Brocchini

**Affiliations:** 1 Department of Construction, Civil Engineering, and Architecture, Polytechnic University of Marche, Ancona, Italy; 2 Formerly with the Department of Pediatric and Congenital Cardiac Surgery and Cardiology, United Hospital, Ancona, Italy

**Keywords:** anomalous coronary anatomy, coronary artery, coronary perfusion autoregulation, *in-vitro* modeling, sudden cardiac death

## Abstract

Sudden cardiac death (SCD) is a leading cause of mortality in young athletes and is frequently linked to congenital anomalies of the coronary arteries, particularly the anomalous origin of the left coronary artery from the right sinus of Valsalva (AOLRS). Despite extensive research, the underlying mechanism remains unclear—especially in cases without structural cardiac abnormalities. In our previous studies, we identified abnormal coronary wave dynamics (“wave pathology”) as a potential factor impairing perfusion in such anomalies. Building on these findings, the present study investigates the hemodynamic consequences of changes in distal coronary resistance in normal and anomalous coronary anatomies. Two *in-vitro* anatomical models—a normal configuration and an AOLRS model with an intramural aortic segment—were tested under rest and exercise conditions in a physiological simulator. By independently varying distal coronary resistances as an experimental representation of distal vasodilation, we observed that decreasing resistance increased flow in the manipulated coronary branch and was accompanied by a modest reduction in mean aortic pressure. In a separate set of tests, decreasing mean aortic pressure while maintaining the coronary resistance settings reduced both LCA and RCA flow, with a stronger pressure-flow dependence during exercise. These results identify a coupling between distal resistance, aortic pressure, and coronary perfusion whose effect becomes more important during exercise. In the anomalous model, this mechanism acts in a coronary circulation in which altered wave propagation and reflection were previously observed, and may add to the hemodynamic vulnerability associated with AOLRS. Its contribution to exercise-induced ischemia and SCD remains to be established.

## 1 Introduction

Coronary artery anomalies (CAAs) are strongly associated with sudden cardiac death (SCD), particularly during or immediately after intense physical exertion (15, 21, 31). Although ischemia is commonly implicated (19), the precise mechanism remains debated. Early hypotheses proposed that the aortic valve leaflets obstruct the coronary ostia during systole, but imaging studies later disproved this (13). Other explanations include compression of the interarterial course by the great vessels during exercise (6, 16), torsion or kinking of the aberrant artery (3–5), and flap-like closure of a slit-shaped ostium during exertion (7, 27). While each mechanism offers partial insight, none fully explains why ischemic events and SCD can occur even in the absence of ostial obstruction, interarterial compression, or structural abnormality (2, 34).

Among these anomalies, the anomalous origin of the left coronary artery from the right sinus of Valsalva (AOLRS) is of particular concern. AOLRS is a form of anomalous aortic origin of a coronary artery (AAOCA), the broader term commonly used for this group of anomalies. Its intramural course within the aortic wall has been linked to altered hemodynamics and elevated SCD risk in young athletes (1, 19). Yet up to 20% of SCD cases show no structural or pathological abnormalities (33, 34), suggesting that the cause may be functional and dynamic—exacerbated by anatomical abnormalities and/or specific cardiac workloads such as intense physical activity.

In our previous work (25), we identified evidence of ***wave pathology***—abnormal wave interactions within the coronary system—that can impair perfusion in anomalous models, particularly under exercise conditions. Building upon these findings, the present study investigates a complementary mechanism: how coronary perfusion autoregulation, acting through distal vascular resistance, interacts with systemic aortic pressure and may further compromise blood supply in anomalous configurations. The pressure–resistance interaction considered here may therefore operate together with the abnormal coronary wave dynamics identified in the anomalous model in our previous study (25), particularly during exercise.

Coronary autoregulation maintains the balance between myocardial oxygen demand and coronary blood supply by adjusting microvascular resistance (9, 13, 17, 18, 22). During exercise, distal vasodilation lowers resistance to meet elevated myocardial oxygen demand. The way these resistance changes interact with aortic pressure and cross-branch flow coupling—especially in anatomically anomalous coronaries—remains poorly understood (8, 9).

To address this issue, we conducted *in-vitro* experiments comparing coronary perfusion behavior in normal and anomalous coronary models under rest and exercise conditions. Controlled changes in distal coronary resistance were used to represent the vasodilatory component of coronary regulation, enabling simultaneous measurement of aortic and coronary pressures and flows. The resistance changes were imposed manually and therefore reproduce only one component of physiological autoregulation. The goal was to clarify how changes in coronary distal resistance and aortic pressure interact to influence coronary perfusion, and whether the anomalous anatomy shows a different response.

The remainder of the paper is organized as follows: Section 2 outlines the role of autoregulation and the rationale for the experimental campaign; Section 3 describes the *in-vitro* models and measurement setup; Section 4 presents and discusses the results; and Section 6 provides concluding remarks.

## 2 Background on coronary autoregulation

The heart has the highest oxygen consumption per gram of tissue of any organ, extracting approximately 70%–80% of delivered oxygen even at rest. This high basal extraction demands a continuously regulated blood supply, governed by a complex autoregulatory system (Table 1). Over the past century, numerous interacting mechanisms have been identified to maintain this balance.

**Table 1:** Factors influencing myocardial oxygen supply and demand.

Myocardial Oxygen Demand	Heart rate	
	Contractility	
	Ventricular Work	
	Internal-potential work	
Myocardial Oxygen Supply	Oxygen carrying capacity	
	Coronary blood flow	**Perfusion Pressure**
		**Vascular Resistance**
		Extravascular compressive forces
		Neurohumoral factors
		Metabolic factors
		Endothelia factors

Coronary perfusion is primarily determined by two physical factors: perfusion pressure and vascular resistance. The latter is dynamically modulated by extravascular compressive forces, myogenic responses, metabolic feedback, neurohormonal signaling, and endothelial regulation. Together, these mechanisms adjust coronary resistance to match myocardial oxygen supply with demand—a process known as pressure–flow autoregulation (13).

Autoregulation adapts continuously to changing metabolic demand (22), integrating both local feedback and sympathetic feedforward control (32). However, despite decades of study, the quantitative coupling between vascular resistance and myocardial metabolism remains incompletely understood (8, 9, 13, 14). Notably, impaired microvascular autoregulation—independent of atherosclerosis—has been recognized as a predictor of cardiac mortality (13).

During physical exertion, mean aortic pressure—the upstream driver of coronary flow—increases only modestly (∼20%–30%), whereas myocardial oxygen demand and coronary flow may rise up to sixfold. This is achieved predominantly through a decrease in coronary vascular resistance, arising from both passive structural dilation and active vasomotor adjustment in small arteries and arterioles (8). Because hemoglobin concentration and oxygen extraction rise only slightly during exertion, the dominant physiological response is an increase in coronary flow. As John Hunter noted in 1794, “*blood goes where it is needed*” (28), yet the precise sensing and feedback mechanisms enabling this adaptation remain partly unresolved (8, 28).

Exercise also modifies ventricular mechanics. The relative prolongation of systole enhances extravascular compressive forces, particularly within the subendocardial layers of the left ventricle, making the fine-tuning of coronary resistance even more critical. The resulting vascular tone reflects a dynamic integration of endothelial, myocardial, and neurohormonal factors, as summarized schematically in Figure 1 (8).

**Figure 1:**
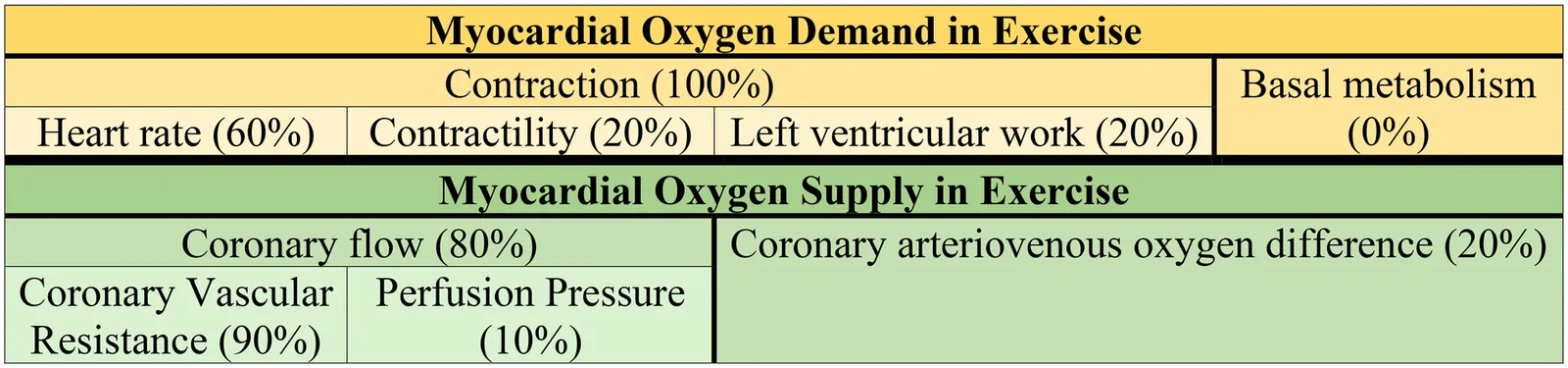
Diagram showing myocardial oxygen supply and demand interactions under exercise conditions.

Experimental studies by Duncker et al. (10) demonstrated these adaptive processes by measuring coronary blood flow across a range of perfusion pressures in exercising dogs, where controlled vasodilation via intra-atrial adenosine infusion revealed how flow regulation depends on both aortic pressure and resistance modulation.

In the present study, the resistance component of coronary autoregulation was represented *in vitro* by systematically varying distal resistance in both the left (LCA) and right (RCA) coronary arteries. Starting from two baseline physiological conditions—previously validated in Mousavi et al. (38) and Mousavi et al. (39)—distal resistances were manually adjusted to represent changes in vascular tone. The model does not reproduce metabolic, myogenic, endothelial, neurohumoral, or baroreflex control. A further set of experiments modulated aortic resistance directly, enabling analysis of the coupling between aortic pressure and coronary perfusion in normal and anomalous anatomical configurations under both rest and exercise loads.

## 3 Methods

### 3.1 Anatomical mock-up models

Two anatomical mock-up models of the human aortic root and coronary arteries were used to replicate *in-vitro* coronary flow under rest and exercise conditions. The models were designed and fabricated as described in Mousavi et al. (38) and Mousavi et al. (39).

The normal model includes the left and right coronary arteries (LCA and RCA) originating from their respective sinuses of Valsalva—left (LS) and right (RS)—as shown in Figures 2A,B. The anomalous model reproduces the anomalous origin of the LCA from the RS, including an intramural segment coursing within the aortic wall (Figure 2C). Key geometrical dimensions are summarized in Table 2. Moreover, further design details on the geometry and material characteristics of the *in-vitro* models are reported in Mousavi et al. (38).

**Figure 2:**
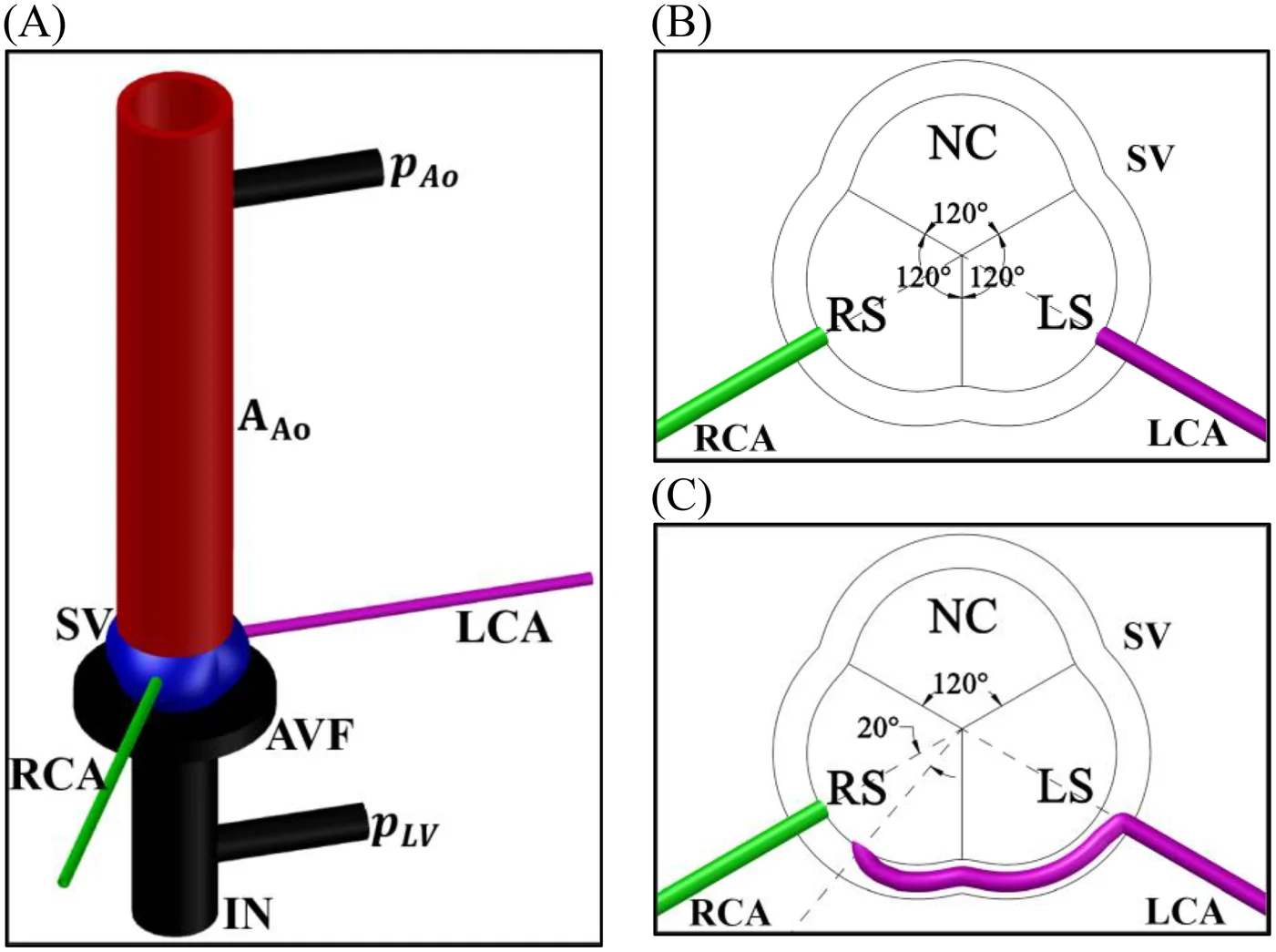
**(A)** Schematic of the *in-vitro* coronary model showing the sinuses of Valsalva (SV), left coronary artery (LCA), right coronary artery (RCA), ascending aorta $\left(A_{\mathrm{Ao}}\right)$, aortic valve flange (AVF), inflow conduit (IN), and pressure ports $\left(p_{\mathrm{LV}}-p_{\mathrm{Ao}}\right)$. **(B)** Normal configuration, where the LCA and RCA originate from the left (LS) and right (RS) sinuses, respectively. **(C)** Anomalous configuration, in which both coronaries arise from the RS and the LCA follows an intramural course within the aortic wall. Adapted from “FIG. 3. **(a)** Schematic of the laboratory model including the aortic sinuses of Valsalva (SV), LCA and RCA, ascending aorta (Asc), aortic valve flange module (AVF), inflow tube (IN), and pressure sensors T-junctions (P1–P2); **(b)** cross section of the Valsalva sinuses (black) with normal origin of the LCA from the left sinuses of Valsalva (LS) and RCA from the right sinuses of Valsalva (RS); and **(c)** anomalous LCA origin from the right sinuses of Valsalva (RS) with an intramural segment coursing within the aortic wall” by Seyyed Mahmoud Mousavi, Gianluca Zitti, Marco Pozzi and Maurizio Brocchini, with the permission of AIP Publishing.

**Table 2:** Geometrical dimensions of the *in-vitro* model.

No	Part	Length (mm)	Diameter (mm)
1	Ascending aorta	235	36
2	Left and right coronary arteries	140	3
3	Sinuses of Valsalva	29	29 (aortic annulus); 36 (sinotubular junction)
4	Inflow conduit	100	29

### 3.2 *In-vitro* setup and experimental procedure

Physiological flow conditions were simulated using a closed-loop *in-vitro* system (Figure 3; see also Mousavi et al. (38); Mousavi et al. (39)). A computer-controlled pulsatile pump circulated fluid through the model, driving flow through the aorta, LCA, and RCA before returning to a reservoir tank. The system included six adjustable resistance valves, two compliance chambers, and a chamber at the distal LCA. Two valves, RRCA and RLCA, allowed independent control of distal coronary resistance. Four pressure transducers recorded left ventricular $\left(\;p_{LV}\right)$, aortic $\left(\;p_{Ao}\right)$, and coronary pressures ($p_{RCA}$ and $p_{LCA}$), while a single flow sensor (FS) sequentially measured the LCA and RCA flow rates $\left(q_{LCA},\;q_{RCA}\right)$. During each resistance-variation series, the pump settings defining the selected rest or exercise condition were kept fixed. The systemic branch was represented by the aortic resistance and compliance elements of the circuit. Mean aortic pressure was therefore an output of the closed-loop circuit when a coronary resistance was changed.

**Figure 3:**
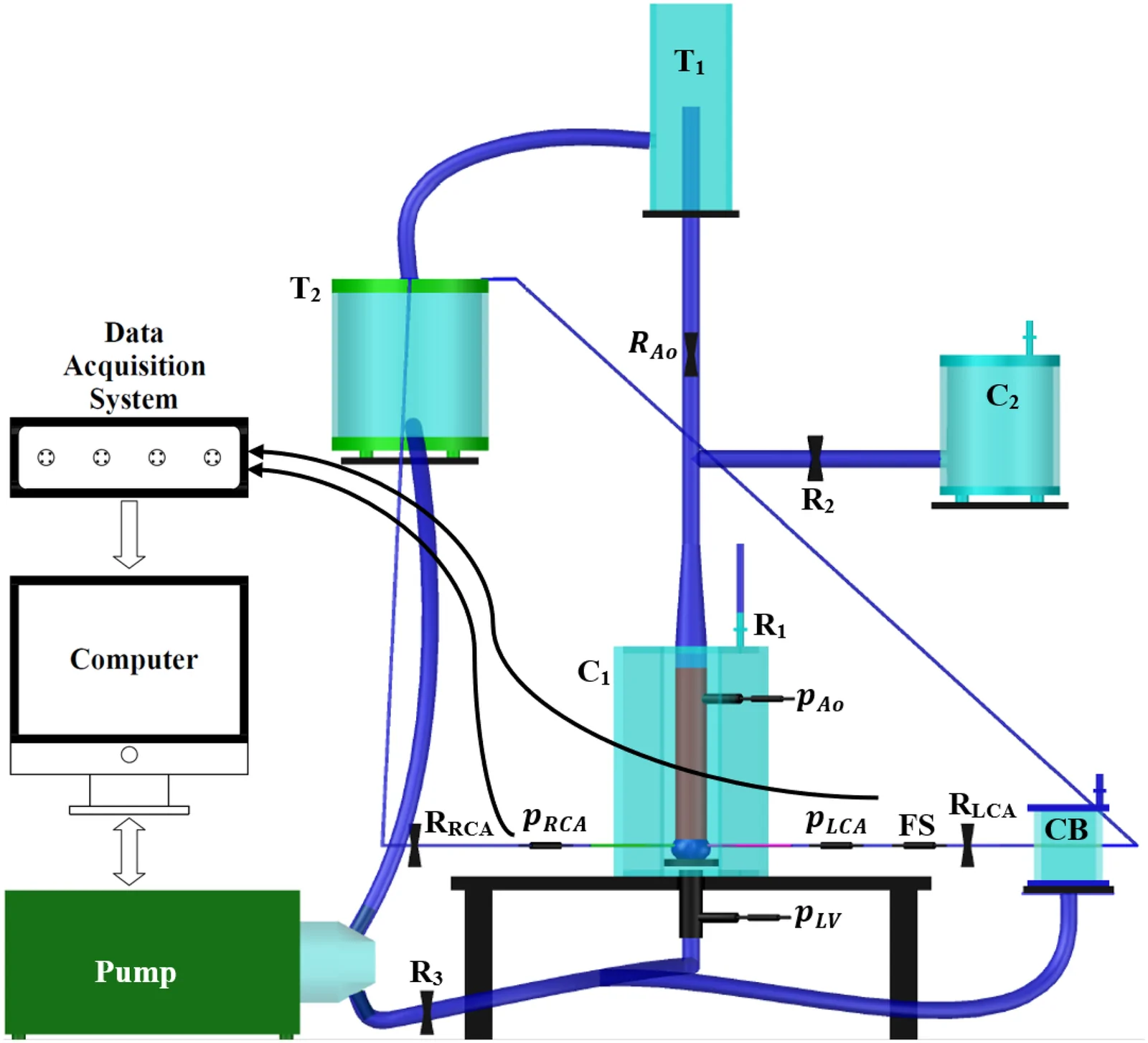
Diagram of the experimental setup. A pulsatile pump provides the driving flow to the coronary model. Flow distribution and pressure conditions are controlled via resistance valves (R_1_–R_3_, R_LCA_, and R_RCA_) and compliance chambers (C_1_, C_2_, CB). Hemodynamic signals are recorded using four pressure transducers ($p_{LCA}$, $p_{RCA}$, $p_{Ao}$, $p_{LV}$) and a flow sensor (FS). Adapted from “FIG. 4. Schematic of the laboratory setup. A computer-controlled pulsatile pump drives the flow inside the laboratory model, where six resistance valves (R1–R6) and three compliance chambers (C1, C2, and CB) regulate the system's fluid dynamics. Additionally, four pressure transducers (P1–P4) and one flowmeter sensor (FS) are used to measure pressure and flow rate values; T1 and T2 are water tanks regulating the reference pressure of the systemic circulation” by Seyyed Mahmoud Mousavi, Gianluca Zitti, Marco Pozzi and Maurizio Brocchini, with the permission of AIP Publishing.

Although the *in-vitro* model replicates human anatomical dimensions, water was used as the working fluid. Because the kinematic viscosity of water is approximately three times lower than that of blood, the pump cardiac cycle was scaled by a factor of three to preserve dynamic similarity—ensuring physiologically relevant Reynolds and Womersley numbers (11, 20, 24).

Two baseline configurations—rest and exercise—were reproduced and validated in our previous work (24). Their main features are summarized in Table 3. Experiments followed the standardized protocol previously described: the system was allowed to stabilize until quasi-periodic steady-state behavior was observed across all sensors. Although perfect periodicity cannot be achieved due to damping and frictional losses (26), cardiovascular systems are typically modeled as quasi-periodic (35).

**Table 3:** Baseline physiological parameters for rest and exercise simulations.

	Cardiovascular variable				Rest		Light exercise	
No	Name	Symbol	Unit	Reference	Lab	Human	Lab	Human
1	Stroke volume	–	*l*	(39)	0.075		0.10	
2	Heart rate	–	$\mathrm{mi}n^{-1}$	(39)	23	70	43	130
3	Cardiac output	Q	$\frac{l}{\min}$	–	1.75	5.25	4.3	13
4	Systolic pressure	–	mmHg	(39)	120		134	
5	Diastolic pressure	–	mmHg	(39)	80		85	
6	Systole/cardiac cycle period	Profile ratio	–	(40)	0.35		0.40	
7	Total coronary flow rate	–	$\frac{l}{\min}$	–	0.070		0.172	
8	Left coronary flow rate	$q_{LCA}$	$\frac{l}{\min}$	(41)	0.042		0.103	
9	Right coronary flow rate	$q_{RCA}$	$\frac{l}{\min}$	(41)	0.028		0.069	

Adapted from “Rest and exercise conditions” by Seyyed Mahmoud Mousavi, Gianluca Zitti and Maurizio Brocchini, licensed under CC BY 4.0.

### 3.3 Experimental sets

From the two baseline conditions (rest and exercise), three sets of experiments were performed:

1. **LCA resistance variation**: distal resistance of the LCA was varied via $R_{\mathrm{LCA}}$.
2. **RCA resistance variation**: distal resistance of the RCA was varied via $R_{\mathrm{RCA}}$.
3. **Aortic pressure variation**: mean aortic pressure was varied via $R_{\mathrm{Ao}}$.

Each test began with baseline pump parameters (Table 3). Resistance valves and compliance chambers (Figure 3) were adjusted until steady periodic signals were achieved. The first two sets imposed controlled changes in distal coronary resistance associated with vasodilation, while the third examined the relation between aortic pressure and coronary perfusion.

In each experiment, coronary flows and pressures (aortic, ventricular, and coronary) were sampled at 2,000 Hz. Data were ensemble-averaged over three cardiac cycles and smoothed using a Savitzky–Golay filter to minimize noise (29, 30). Each operating point corresponds to one stabilized recording. Repeatability of the same experimental platform was assessed in our previous work (24) using four independent runs at identical settings; the coefficients of variation were 1.7% for LV pressure, 0.37% for aortic pressure, 0.44% for LCA pressure, and 0.39% for RCA pressure. These repeatability tests refer to the platform and are not replicate measurements for every operating point of the present sweeps. For the pressure-variation series, mean coronary flow was fitted against mean aortic pressure by ordinary least squares. Slopes are reported with 95% confidence intervals, and normal and anomalous slopes were compared using a pressure-by-anatomy interaction term. A two-sided *p* < 0.05 was used for the comparison.

### 3.4 Calculation of distal resistance

Distal coronary resistances were quantified by adjusting valve openings and applying the pressure–flow relationship commonly used in Windkessel-type models (23, 37) —Equations (1) and (2):

$${\bar{R}}_{LCA}=\frac{{\bar{\Delta p}}_{LCA}}{{\bar{q}}_{LCA}}$$ (1)

$${\bar{R}}_{RCA}=\frac{{\bar{\Delta p}}_{RCA}}{{\bar{q}}_{RCA}}$$ (2)

where ${\bar{\Delta p}}_{LCA}$ and ${\bar{\Delta p}}_{RCA}$ are the time-averaged pressure drops across the LCA and RCA, respectively (measured coronary pressures $p_{LCA}$, $p_{RCA}$ minus the atmospheric outlet reference, assumed 0 mmHg), and ${\bar{q}}_{LCA}$ and ${\bar{q}}_{RCA}$ are the corresponding time-averaged flow rates. All averages were computed over a complete cardiac cycle. For clarity, Table 4 summarizes the variables and acronyms used throughout the manuscript.

**Table 4:** List of variables and acronyms used throughout the manuscript.

Acronym/Symbol	Variable Definition
A_Ao_	Ascending aorta
AVF	Aortic valve flange module
FS	Flow sensor
IN	Inflow conduit
LCA	Left coronary artery
LS	Left sinuses of Valsalva
RCA	Right coronary artery
RS	Right sinuses of Valsalva
SV	Sinuses of Valsalva
$p_{Ao}$	Pressure of aorta
$p_{LCA}$	Pressure of LCA
$p_{LV}$	Pressure of left ventricle
$p_{RCA}$	Pressure of RCA
$q_{LCA}$	Flow rate of LCA
$q_{RCA}$	Flow rate of RCA

## 4 Results and discussion

We first consider the two baseline physiological tests. Moving from rest to exercise conditions, baseline levels of coronary perfusion required adjustment of the distal resistance valves. This is consistent with the established role of distal vascular resistance in the increase of coronary flow during exercise (9). In the present experiments the resistance values are imposed settings.

Results summarized in Table 5 show that, in the LCA of both models, transition from rest to exercise increased mean coronary flow rates by more than 130%, whereas mean aortic pressure rose by only ≈12%. The baseline exercise LCA flow was 99.45 mL/min in the normal model and 104.04 mL/min in the anomalous model. The anomalous model therefore did not show a lower mean LCA flow at the calibrated exercise baseline.

**Table 5:** Time-averaged aortic pressure and coronary flow rates in normal and anomalous models under rest and exercise conditions.

	${\bar{\;p}}_{Ao}(\mathrm{mmHg})$	${\bar{q}}_{LCA}(\mathrm{mL}/\min)$	${\bar{R}}_{LCA}\left(\mathrm{mmHg}\;\times \;\frac{\min}{\mathrm{mL}}\right)$	${\bar{q}}_{RCA}(\mathrm{mL}/\min)$	${\bar{R}}_{RCA}\left(\mathrm{mmHg}\times \frac{\min}{\mathrm{mL}}\right)$
Normal model					
Rest	98.70	42.75	2.28	30.76	3.16
Exercise	111.05	99.45	1.04	67.06	1.63
$\frac{\mathrm{Exercise}-\mathrm{Rest}}{\mathrm{Rest}}\times 100$	12.51%	132.63%	−54.39%	118.01%	−48.42%
Anomalous model					
Rest	98.51	41.74	2.33	32.30	3.04
Exercise	110.35	104.04	1.01	67.20	1.61
$\frac{\mathrm{Exercise}-\mathrm{Rest}}{\mathrm{Rest}}\times 100$	12.02%	149.26%	−56.65%	108.05%	−47.04%

Motivated by these baseline findings, we conducted two series of experiments to examine how changes in coronary resistance influence global hemodynamics. The first and second experimental sets independently varied (i) mean LCA distal resistance and (ii) mean RCA distal resistance from the physiological baseline under both rest and exercise conditions. The time-averaged results (pressures, flow rates, and computed resistances) are reported in Supplementary Appendix A and discussed below.

To compare results quantitatively, we computed the percentage change of each time-averaged variable relative to its baseline value using Equation (3):

$$\Delta \left({\bar{\Phi }}_{i}\right)=\frac{{\bar{\Phi }}_{i}-{\bar{\Phi }}_{B}}{{\bar{\Phi }}_{B}}\times 100$$ (3)

where ${\bar{\Phi }}_{i}$ is the time-averaged value of a given variable (pressure, flow rate, or resistance) in the ith test, and ${\bar{\Phi }}_{B}$ is its baseline value. In the tables (Supplementary Appendix B), we report the two extreme cases of each coronary resistance variation: (i) maximum increase in mean coronary resistance ($\max\Delta {\bar{R}}_{LCA}$ or $\max\Delta {\bar{R}}_{RCA}$) and (ii) maximum decrease ($\min\Delta {\bar{R}}_{LCA}$ or $\min\Delta {\bar{R}}_{RCA}$), together with the corresponding percentage changes in mean pressures and flow rates.

### 4.1 Effects of distal resistance variation

Figure 4 illustrates how varying LCA resistance affects mean pressure (panels A, C) and RCA flow rate (panels B, D) in the normal (A, B) and anomalous (C, D) models during rest. A decrease in LCA resistance consistently reduced pressures across all measurement sites, while the RCA flow showed a non-uniform response. Supplementary Appendix B also shows the direct response of the manipulated branch. From the baseline to the lowest finite LCA resistance, LCA flow increased by 205% and 254% at rest in the normal and anomalous models, and by 21% and 23% during exercise. Mean aortic pressure decreased by 3.1%, 4.1%, 0.2%, and 0.1% over the same changes. The RCA-resistance tests showed the same direct relation between lower branch resistance and higher ipsilateral flow.

**Figure 4:**
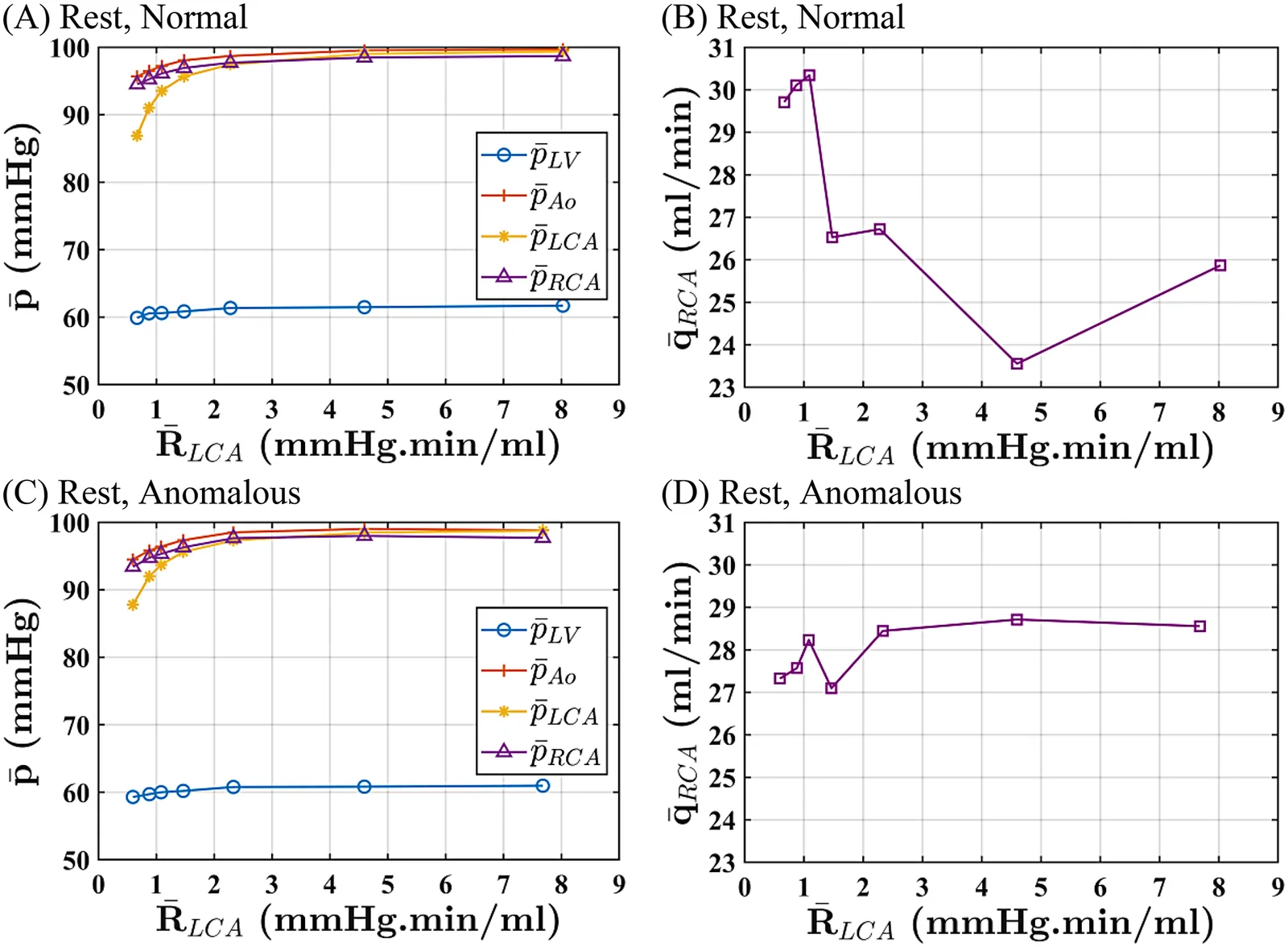
Effects of varying mean LCA resistance $\left({\bar{R}}_{LCA}\right)$ under rest conditions. Panels **(A)** and **(B)** show results for the normal model: **(A)** time-averaged pressures (${\bar{\;p}}_{LV},\;{\bar{\;p}}_{Ao},\;{\bar{\;p}}_{LCA}$, and ${\bar{\;p}}_{RCA}$) vs. mean LCA resistance, and **(B)** mean RCA flow rate $\left({\bar{q}}_{RCA}\right)$ vs. mean LCA resistance. Panels **(C)** and **(D)** show the corresponding results for the anomalous model: **(C)** time-averaged pressures vs. mean LCA resistance, and **(D)** mean RCA flow rate vs. mean LCA resistance.

These findings are consistent with our previous results (25), where distinct wave dynamics were observed between the normal and anomalous models. The resistance-variation experiments show an increase in ipsilateral coronary flow when distal resistance is reduced. At the same time, mean aortic pressure decreases modestly in the closed-loop circuit. The possible effect of this pressure change on coronary flow was examined separately.

Further quantitative analysis (Supplementary Appendix B) showed that mean aortic pressure $\left({\bar{\;p}}_{Ao}\right)$ consistently decreased whenever either coronary resistance was reduced. Coronary perfusion is strongly influenced by aortic pressure (12), with phasic ventricular contractions generating distinct systolic and diastolic flow components. This observation prompted the additional pressure-variation experiments described below.

### 4.2 Effect of aortic pressure on coronary perfusion

To isolate this pressure-flow relation, 32 additional operating points were obtained by directly varying mean aortic pressure—through adjustments to valve (Figure 3)—while keeping the coronary resistance settings fixed. The results, summarized in Supplementary Appendix C, show how changes in aortic pressure influence LCA and RCA perfusion under rest and exercise conditions.

Figure 5 shows that decreasing mean aortic pressure at fixed coronary resistance is associated with a consistent decline in both LCA and RCA flow rates. Linear regression analysis yielded pressure-flow slopes (*α*) quantifying the sensitivity of each coronary branch to pressure variations.

**Figure 5:**
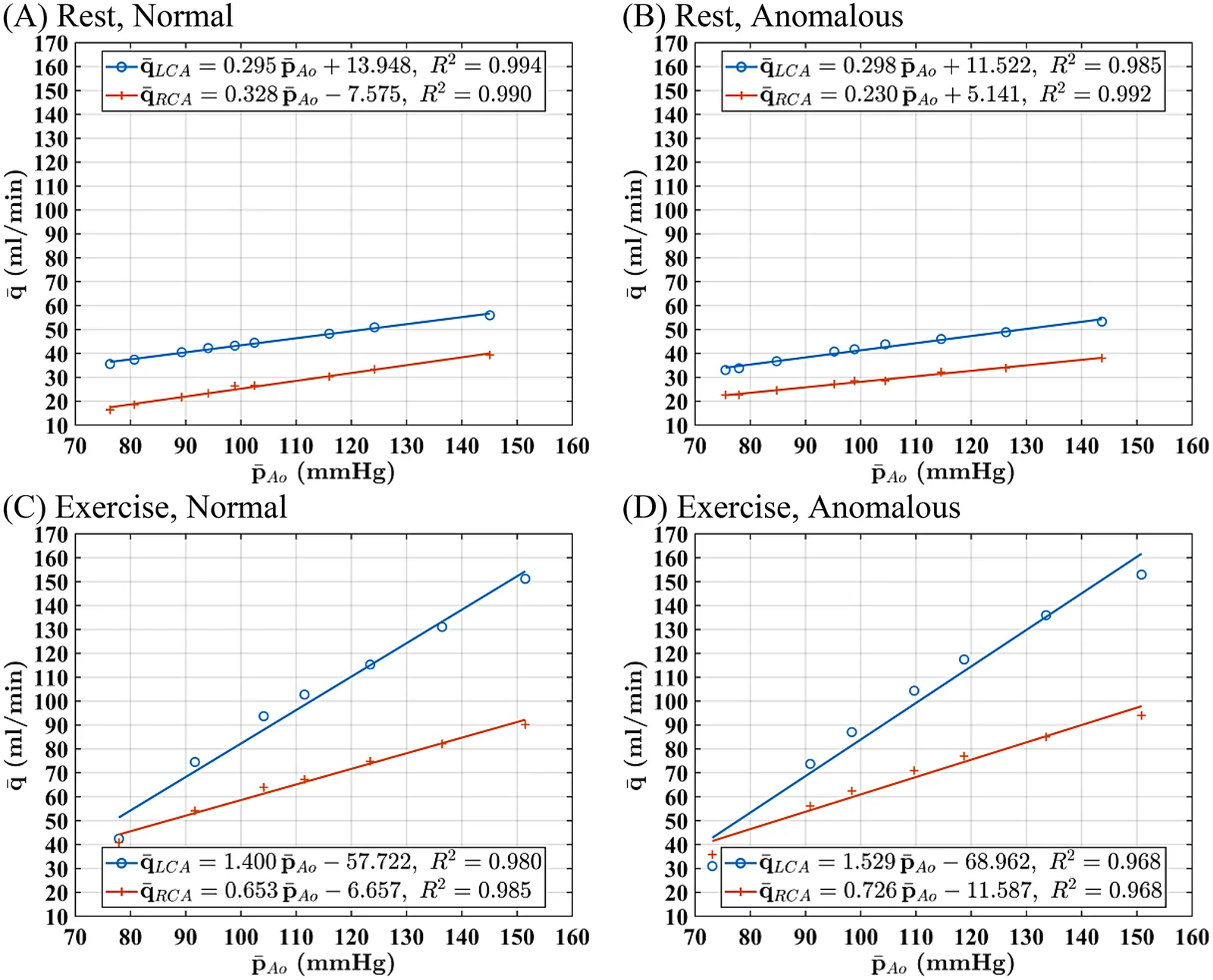
Effects of mean aortic pressure on coronary perfusion. Panels **(A)** and **(B)** show the relationship between mean aortic pressure and measured mean LCA and RCA flow rates during rest in the normal and anomalous models, respectively. Panels **(C)** and **(D)** present the corresponding results during exercise for the normal and anomalous models.

For the LCA under rest, *α* was 0.295 mL min^−1^ mmHg^−1^ (95% CI 0.274–0.315; R^2^ = 0.994) in the normal model and 0.298 mL min^−1^ mmHg^−1^ (95% CI 0.265–0.331; R^2^ = 0.985) in the anomalous model. The difference between the two slopes was not significant (*p* = 0.846).

During exercise (Figures 5C,D), the LCA pressure-flow slopes increased to 1.400 mL min^−1^ mmHg^−1^ (95% CI 1.167–1.632; R^2^ = 0.980) in the normal model and 1.529 mL min^−1^ mmHg^−1^ (95% CI 1.211–1.847; R^2^ = 0.968) in the anomalous model. Their difference was not significant (*p* = 0.422). In both models the exercise slope was higher than the corresponding rest slope (*p* < 0.001).

For the RCA, the rest slopes were 0.328 mL min^−1^ mmHg^−1^ (95% CI 0.298–0.358; R^2^ = 0.990) in the normal model and 0.230 mL min^−1^ mmHg^−1^ (95% CI 0.212–0.248; R^2^ = 0.992) in the anomalous model; the difference was significant (*p* < 0.001), with the lower slope in the anomalous model. During exercise, the slopes were 0.653 mL min^−1^ mmHg^−1^ (95% CI 0.561–0.745; R^2^ = 0.985) and 0.726 mL min^−1^ mmHg^−1^ (95% CI 0.575–0.877; R^2^ = 0.968), respectively, with no significant difference between anatomies (*p* = 0.317).

Overall, these experiments confirm that coronary perfusion is tightly coupled to mean aortic pressure and that pressure-flow sensitivity increases markedly during exercise. For the LCA, the normal and anomalous slopes were not significantly different at either workload. The pressure–flow slope describes the response of mean coronary flow to mean aortic pressure at fixed distal resistance. The anomalous model also exhibits the wave-propagation abnormalities reported in (25), which are not represented by this parameter. The pressure sensitivity measured here and the previously observed wave dynamics therefore describe different components of coronary perfusion under exercise conditions.

### 4.3 Physiological interpretation

During intense exertion, myocardial oxygen demand rises sharply due to increased heart rate, contractility, and ventricular workload. Coronary vasodilation reduces distal resistance to increase blood supply. In the resistance-variation experiments, lowering distal resistance increased flow in the manipulated branch and was accompanied by a modest decrease in mean aortic pressure. The separate pressure-variation experiments showed that lower aortic pressure reduces coronary flow at fixed distal resistance. Together, these observations suggest that a decrease in aortic pressure could reduce part of the flow gain produced by distal vasodilation. The full physiological feedback system is outside the present *in-vitro* setup; Figure 6 shows this possible interaction schematically.

**Figure 6:**
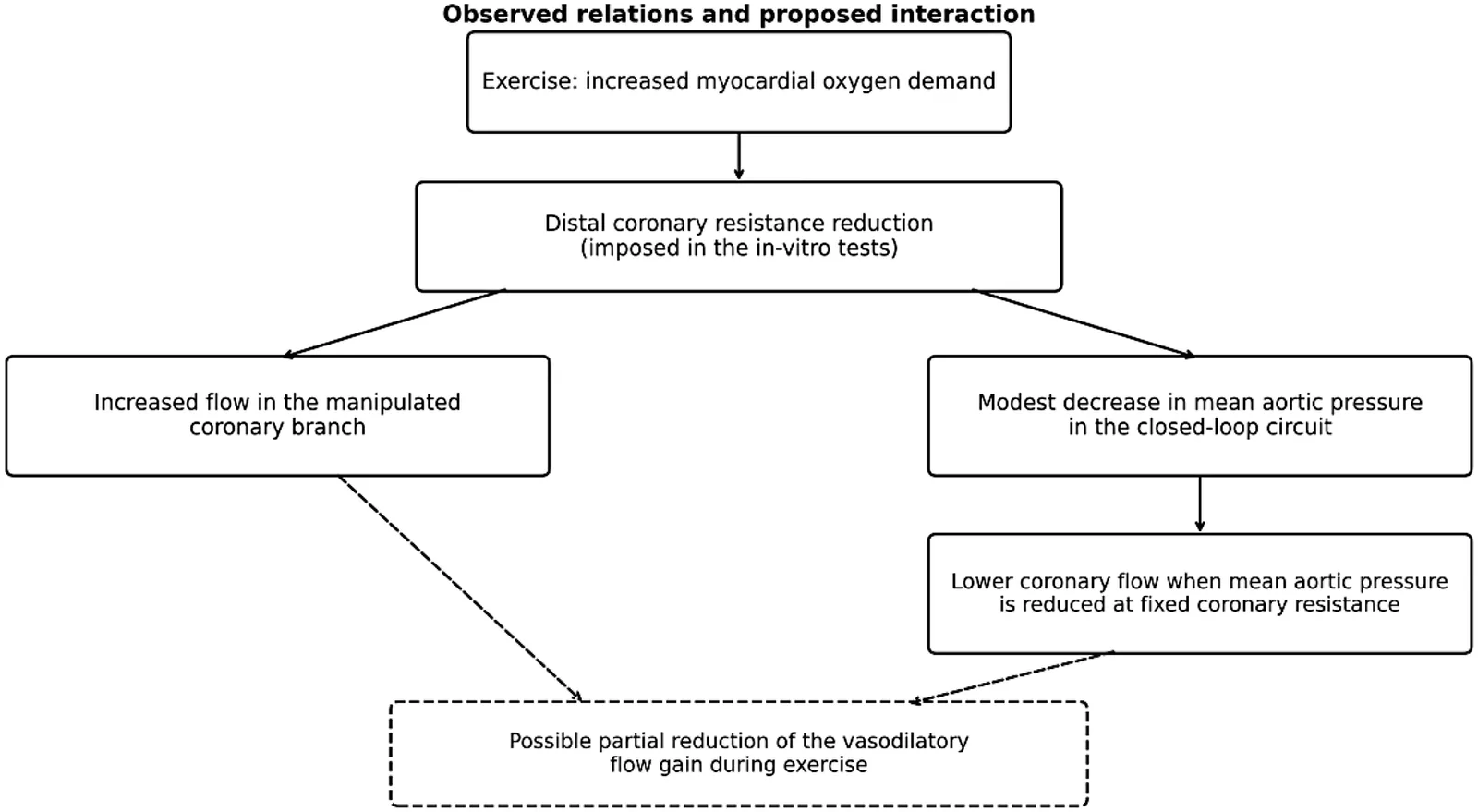
Schematic summary of the experimental observations and their relation to the anomalous coronary configuration. Solid arrows show relations measured in the present resistance- and pressure-variation tests. Altered wave dynamics in the anomalous LCA were demonstrated in our previous study (25). Dashed arrows indicate the proposed interaction of these mechanisms during exercise.

In both models, the pressure-flow slope increased substantially during exercise. For the LCA, the present data show similar slopes in the normal and anomalous models. The anomalous geometry remains important in this interpretation. In our previous experiments (25), the same anomalous LCA showed changes in wave polarity, amplification of secondary wave-intensity peaks, and an increase in wave speed during exercise. The pressure–resistance coupling identified here may act together with these wave disturbances during exertion, when the pressure–flow sensitivity is also markedly increased. The present experiments do not determine the relative contribution of these effects, but they indicate that more than one dynamic mechanism may affect coronary perfusion in the anomalous configuration during exercise.

The models exclude the pulmonary artery and therefore do not reproduce possible external compression of an interarterial coronary course. No surgically corrected geometry was tested. Mechanical compression, ostial deformation, wave dynamics, and pressure-resistance coupling may contribute in different proportions in patients, and the present experiments cannot establish their relative importance.

In summary, these *in-vitro* experiments show a coupling between coronary distal resistance, mean aortic pressure, and coronary flow. The resistance-variation tests produced higher ipsilateral flow together with a modest fall in mean aortic pressure, while the separate pressure-variation tests showed lower coronary flow when aortic pressure was reduced. The pressure-flow dependence was stronger during exercise. The present results therefore add pressure–resistance coupling to the wave-dynamic abnormalities previously identified in the anomalous model. During exercise, the simultaneous action of these effects may reduce the ability of the anomalous coronary circulation to maintain adequate perfusion. The magnitude of this combined effect *in vivo* remains to be established.

## 5 Limitations

The experiments were designed to isolate the interaction between distal coronary resistance, aortic pressure, and coronary flow under controlled rest and exercise conditions. Distal resistance is adjusted manually, while metabolic, myogenic, endothelial, neurohumoral, sympathetic, and baroreflex regulation are not represented. Ventricular loading of the LCA is reproduced through the coronary box (24), although the full spatial distribution of myocardial contraction and extravascular compression is not included. The systemic circulation is represented by lumped resistance and compliance elements; therefore, the observed decrease in mean aortic pressure reflects the response of the present closed-loop system and its magnitude *in vivo* may differ because of systemic compensatory mechanisms.

The pulmonary artery and possible external compression of the interarterial coronary course are not reproduced, and a single AOLRS geometry with an intramural segment was examined. Repeatability of the experimental platform was previously verified in four independent runs (24), with pressure coefficients of variation below 2%. Individual operating points of the present sweeps were not independently repeated. The model measures coronary pressure and flow but does not directly assess myocardial oxygen balance, ischemia, arrhythmia, or SCD, and a surgically corrected geometry was not tested.

## 6 Concluding remarks

Sudden cardiac death (SCD) in young individuals is often associated with congenital coronary anomalies, particularly the anomalous origin of the left coronary artery (LCA) from the right sinus of Valsalva with an intramural aortic course. Our previous study (25) showed that this geometry alters coronary wave dynamics, with the clearest differences occurring during exercise. The present experiments examine an additional hemodynamic interaction involving coronary distal resistance and aortic pressure.

Reductions in distal coronary resistance increased flow in the manipulated branch and were accompanied by a modest decrease in mean aortic pressure. When mean aortic pressure was varied independently, both the LCA and RCA showed reduced flow as pressure decreased, and the pressure–flow dependence increased markedly during exercise.

The LCA pressure–flow slopes did not differ significantly between the normal and anomalous models. In the anomalous model, however, this pressure–resistance interaction occurs in a coronary circulation already characterized by abnormal wave dynamics. The two effects may act simultaneously during exercise and may reduce the margin available for maintaining coronary perfusion. This provides a possible functional contribution to the increased risk associated with AOLRS.

The experiments do not establish a direct causal link with myocardial ischemia, arrhythmia or SCD. Patient-specific studies and experiments with surgically corrected geometries are required to determine the clinical importance of the proposed interaction and its modification by surgical treatment.

## Data Availability

The original contributions presented in the study are included in the article/Supplementary Material, further inquiries can be directed to the corresponding author.
