# Supplementary material for: The role of vascular resistance on coronary perfusion: differences between healthy and anomalous coronary origin under rest and exercise conditions
Source: Front Cardiovasc Med. 2026 Oct 13;13:1881521. doi: 10.3389/fcvm.2026.1881521 (PMC13710293; doi:10.3389/fcvm.2026.1881521)
Supplement: Supplementary file 1 [file Supplementaryfile1.docx]

# 1 Appendix A

This appendix presents the plots associated with variations in distal resistances of the LCA and RCA in both the normal and anomalous models under rest and exercise conditions.

## 1.1 Results of LCA resistance variation

| (A) Exercise, Normal   | (B) Exercise, Normal   |
| --- | --- |
| (C) Exercise, Anomalous   | (D) Exercise, Anomalous   |

Figure 1. Effects of varying mean LCA resistance ($\bar{R}_{LCA}$) under exercise conditions. Panels (A) and (B) show results for the normal model: (A) time-averaged pressures ($\bar{p}_{LV}, \bar{p}_{Ao}, \bar{p}_{LCA}$, and $\bar{p}_{RCA}$) versus mean LCA resistance, and (B) mean RCA flow rate ($\bar{q}_{RCA}$) versus mean LCA resistance. Panels (C) and (D) show the corresponding results for the anomalous model: (C) time-averaged pressures versus mean LCA resistance, and (D) mean RCA flow rate versus mean LCA resistance.

## 1.2 Results of RCA resistance variation

| (A) Rest, Normal   | (B) Rest, Normal   |
| --- | --- |
| (C) Rest, Anomalous   | (D) Rest, Anomalous   |

Figure 2. Effects of varying mean RCA resistance ($\bar{R}_{RCA}$) under rest conditions. Panels (A) and (B) show results for the normal model: (A) time-averaged pressures ($\bar{p}_{LV}, \bar{p}_{Ao}, \bar{p}_{LCA}$, and $\bar{p}_{RCA}$) versus mean RCA resistance, and (B) mean LCA flow rate ($\bar{q}_{LCA}$) versus mean RCA resistance. Panels (C) and (D) show the corresponding results for the anomalous model: (C) time-averaged pressures versus mean RCA resistance, and (D) mean LCA flow rate versus mean RCA resistance.

| (A) Exercise, Normal   | (B) Exercise, Normal   |
| --- | --- |
| (C) Exercise, Anomalous   | (D) Exercise, Anomalous   |

Figure 3. Effects of varying mean RCA resistance ($\bar{R}_{RCA}$) under exercise conditions. Panels (A) and (B) show results for the normal model: (A) time-averaged pressures ($\bar{p}_{LV}, \bar{p}_{Ao}, \bar{p}_{LCA}$, and $\bar{p}_{RCA}$) versus mean RCA resistance, and (B) mean LCA flow rate ($\bar{q}_{LCA}$) versus mean RCA resistance. Panels (C) and (D) show the corresponding results for the anomalous model: (C) time-averaged pressures versus mean RCA resistance, and (D) mean LCA flow rate versus mean RCA resistance.

# 2 Appendix B

This appendix reports the time-averaged pressures ($\bar{p}_{LCA}, \bar{p}_{RCA}, \bar{p}_{LV}$, and $\bar{p}_{Ao}$), coronary flow rates ($\bar{q}_{LCA}$ and $\bar{q}_{RCA}$), and resistance values ($\bar{R}_{LCA}$ and $\bar{R}_{RCA}$) obtained during LCA and RCA resistance-variation experiments. In each table, the data row highlighted in blue corresponds to the baseline physiological condition (rest or exercise), reproducing the reference values listed in Table 3 of the main manuscript.

## 2.1 LCA resistance variation

Table 1. Time-averaged pressures ($\bar{p}_{LCA}, \bar{p}_{RCA}, \bar{p}_{LV}$, and $\bar{p}_{Ao}$, in $mmHg$), coronary flow rates ($\bar{q}_{LCA}$ and $\bar{q}_{RCA}$, in $ml/min$), and resistance values ($\bar{R}_{LCA}$ and $\bar{R}_{RCA}$, in $mmHg\times min/ml$) during **LCA** resistance variation in the **normal model** under **rest** conditions.

| ${\bar{\boldsymbol{p}}}_{\boldsymbol{LV}}$ | ${\bar{\boldsymbol{p}}}_{\boldsymbol{Ao}}$ | ${\bar{\boldsymbol{p}}}_{\boldsymbol{LCA}}$ | ${\bar{\boldsymbol{p}}}_{\boldsymbol{RCA}}$ | ${\bar{\boldsymbol{q}}}_{\boldsymbol{LCA}}$ | ${\bar{\boldsymbol{q}}}_{\boldsymbol{RCA}}$ | ${\bar{\boldsymbol{R}}}_{\boldsymbol{LCA}}$ | ${\bar{\boldsymbol{R}}}_{\boldsymbol{RCA}}$ |
| --- | --- | --- | --- | --- | --- | --- | --- |
| 59.91 | 95.66 | 86.90 | 94.50 | 130.25 | 29.71 | 0.67 | 3.18 |
| 60.57 | 96.48 | 91.03 | 95.25 | 104.27 | 30.11 | 0.87 | 3.16 |
| 60.61 | 97.20 | 93.54 | 96.12 | 85.60 | 30.34 | 1.09 | 3.17 |
| 60.85 | 98.06 | 95.67 | 96.93 | 64.72 | 26.53 | 1.48 | 3.65 |
| 61.36 | 98.70 | 97.44 | 97.70 | 42.75 | 26.72 | 2.28 | 3.66 |
| 61.48 | 99.53 | 99.01 | 98.47 | 21.54 | 23.56 | 4.60 | 4.18 |
| 61.71 | 99.70 | 99.35 | 98.68 | 12.38 | 25.87 | 8.03 | 3.82 |
| 62.20 | 99.77 | 99.69 | 98.69 | 0.00 | 25.20 | - | 3.92 |

Table 2. Percentage changes in mean pressure and flow values of the **normal** **model** under **rest** conditions, corresponding to maximum and minimum variations of **LCA** distal resistance ($\Delta\bar{R}_{LCA}$) relative to the baseline condition.

| LCA | $\Delta\bar{p}_{LV}\left( \% \right)$ | $\Delta\bar{p}_{Ao}\left( \% \right)$ | $\Delta\bar{p}_{LCA}\left( \% \right)$ | $\Delta\bar{p}_{RCA}\left( \% \right)$ | $\Delta\bar{q}_{RCA}\left( \% \right)$ |
| --- | --- | --- | --- | --- | --- |
| $\max\Delta\bar{R}_{LCA}=252.27 \%$ | $1.37$ | $1.08$ | $2.31$ | $1.01$ | $-5.67$ |
| $\min\Delta\bar{R}_{LCA}=-70.73 \%$ | $-2.38$ | $-3.08$ | $-10.82$ | $-3.28$ | $11.19$ |

Table 3. Time-averaged pressures ($\bar{p}_{LCA}, \bar{p}_{RCA}, \bar{p}_{LV}$, and $\bar{p}_{Ao}$, in $mmHg$), coronary flow rates ($\bar{q}_{LCA}$ and $\bar{q}_{RCA}$, in $ml/min$), and resistance values ($\bar{R}_{LCA}$ and $\bar{R}_{RCA}$, in $mmHg\times min/ml$) during **LCA** resistance variation in the **anomalous model** under **rest** conditions.

| ${\bar{\boldsymbol{p}}}_{\boldsymbol{LV}}$ | ${\bar{\boldsymbol{p}}}_{\boldsymbol{Ao}}$ | ${\bar{\boldsymbol{p}}}_{\boldsymbol{LCA}}$ | ${\bar{\boldsymbol{p}}}_{\boldsymbol{RCA}}$ | ${\bar{\boldsymbol{q}}}_{\boldsymbol{LCA}}$ | ${\bar{\boldsymbol{q}}}_{\boldsymbol{RCA}}$ | ${\bar{\boldsymbol{R}}}_{\boldsymbol{LCA}}$ | ${\bar{\boldsymbol{R}}}_{\boldsymbol{RCA}}$ |
| --- | --- | --- | --- | --- | --- | --- | --- |
| 59.30 | 94.48 | 87.77 | 93.40 | 147.65 | 27.32 | 0.59 | 3.42 |
| 59.71 | 95.80 | 92.00 | 94.74 | 104.50 | 27.57 | 0.88 | 3.44 |
| 60.00 | 96.40 | 93.70 | 95.32 | 86.58 | 28.23 | 1.08 | 3.38 |
| 60.20 | 97.35 | 95.60 | 96.28 | 65.28 | 27.10 | 1.46 | 3.55 |
| 60.78 | 98.51 | 97.32 | 97.65 | 41.74 | 28.44 | 2.33 | 3.43 |
| 60.82 | 99.02 | 98.46 | 97.98 | 21.43 | 28.71 | 4.60 | 3.41 |
| 60.97 | 98.82 | 98.76 | 97.69 | 12.86 | 28.56 | 7.68 | 3.42 |
| 61.03 | 99.31 | 99.32 | 98.23 | 0.00 | 28.72 | - | 3.42 |

Table 4. Percentage changes in mean pressure and flow values of the **anomalous** **model** under **rest** conditions, corresponding to maximum and minimum variations of **LCA** distal resistance ($\Delta\bar{R}_{LCA}$) relative to the baseline condition.

| LCA | $\Delta\bar{p}_{LV}\left( \% \right)$ | $\Delta\bar{p}_{Ao}\left( \% \right)$ | $\Delta\bar{p}_{LCA}\left( \% \right)$ | $\Delta\bar{p}_{RCA}\left( \% \right)$ | $\Delta\bar{q}_{RCA}\left( \% \right)$ |
| --- | --- | --- | --- | --- | --- |
| $\max\Delta\bar{R}_{LCA}=229.39 \%$ | $0.43$ | $0.81$ | $2.06$ | $0.60$ | $0.97$ |
| $\min\Delta\bar{R}_{LCA}=-74.50 \%$ | $-2.43$ | $-4.09$ | $-9.81$ | $-4.35$ | $-3.94$ |

Table 5. Time-averaged pressures ($\bar{p}_{LCA}, \bar{p}_{RCA}, \bar{p}_{LV}$, and $\bar{p}_{Ao}$, in $mmHg$), coronary flow rates ($\bar{q}_{LCA}$ and $\bar{q}_{RCA}$, in $ml/min$), and resistance values ($\bar{R}_{LCA}$ and $\bar{R}_{RCA}$, in $mmHg\times min/ml$) during **LCA** resistance variation in the **normal model** under **exercise** conditions.

| ${\bar{\boldsymbol{p}}}_{\boldsymbol{LV}}$ | ${\bar{\boldsymbol{p}}}_{\boldsymbol{Ao}}$ | ${\bar{\boldsymbol{p}}}_{\boldsymbol{LCA}}$ | ${\bar{\boldsymbol{p}}}_{\boldsymbol{RCA}}$ | ${\bar{\boldsymbol{q}}}_{\boldsymbol{LCA}}$ | ${\bar{\boldsymbol{q}}}_{\boldsymbol{RCA}}$ | ${\bar{\boldsymbol{R}}}_{\boldsymbol{LCA}}$ | ${\bar{\boldsymbol{R}}}_{\boldsymbol{RCA}}$ |
| --- | --- | --- | --- | --- | --- | --- | --- |
| 66.04 | 110.81 | 101.21 | 108.97 | 120.51 | 65.69 | 0.84 | 1.66 |
| 66.25 | 111.05 | 103.87 | 109.10 | 99.45 | 66.05 | 1.04 | 1.65 |
| 66.54 | 111.30 | 105.49 | 108.77 | 88.75 | 67.53 | 1.19 | 1.61 |
| 66.24 | 111.50 | 107.22 | 109.64 | 75.70 | 67.95 | 1.42 | 1.61 |
| 66.82 | 111.88 | 108.51 | 109.98 | 62.59 | 68.55 | 1.73 | 1.60 |
| 67.10 | 112.96 | 112.39 | 110.98 | 0.00 | 69.30 | - | 1.60 |

Table 6. Percentage changes in mean pressure and flow values of the **normal** **model** under **exercise** conditions, corresponding to maximum and minimum variations of **LCA** distal resistance ($\Delta\bar{R}_{LCA}$) relative to the baseline condition.

| LCA | $\Delta\bar{p}_{LV}\left( \% \right)$ | $\Delta\bar{p}_{Ao}\left( \% \right)$ | $\Delta\bar{p}_{LCA}\left( \% \right)$ | $\Delta\bar{p}_{RCA}\left( \% \right)$ | $\Delta\bar{q}_{RCA}\left( \% \right)$ |
| --- | --- | --- | --- | --- | --- |
| $\max\Delta\bar{R}_{LCA}=66.60 \%$ | $1.29$ | $1.72$ | $8.21$ | $1.72$ | $4.92$ |
| $\min\Delta\bar{R}_{LCA}=-19.58 \%$ | $-0.31$ | $-0.21$ | $-2.55$ | $-0.12$ | $-0.54$ |

Table 7. Time-averaged pressures ($\bar{p}_{LCA}, \bar{p}_{RCA}, \bar{p}_{LV}$, and $\bar{p}_{Ao}$, in $mmHg$), coronary flow rates ($\bar{q}_{LCA}$ and $\bar{q}_{RCA}$, in $ml/min$), and resistance values ($\bar{R}_{LCA}$ and $\bar{R}_{RCA}$, in $mmHg\times min/ml$) during **LCA** resistance variation in the **anomalous model** under **exercise** conditions.

| ${\bar{\boldsymbol{p}}}_{\boldsymbol{LV}}$ | ${\bar{\boldsymbol{p}}}_{\boldsymbol{Ao}}$ | ${\bar{\boldsymbol{p}}}_{\boldsymbol{LCA}}$ | ${\bar{\boldsymbol{p}}}_{\boldsymbol{RCA}}$ | ${\bar{\boldsymbol{q}}}_{\boldsymbol{LCA}}$ | ${\bar{\boldsymbol{q}}}_{\boldsymbol{RCA}}$ | ${\bar{\boldsymbol{R}}}_{\boldsymbol{LCA}}$ | ${\bar{\boldsymbol{R}}}_{\boldsymbol{RCA}}$ |
| --- | --- | --- | --- | --- | --- | --- | --- |
| 67.23 | 110.20 | 102.97 | 109.47 | 128.18 | 71.86 | 0.80 | 1.52 |
| 67.24 | 110.35 | 105.12 | 109.51 | 104.04 | 66.49 | 1.01 | 1.65 |
| 66.91 | 109.53 | 105.48 | 107.20 | 89.48 | 70.47 | 1.18 | 1.52 |
| 66.73 | 110.16 | 106.98 | 107.86 | 76.71 | 69.72 | 1.39 | 1.55 |
| 66.85 | 110.28 | 107.84 | 107.88 | 63.70 | 70.73 | 1.69 | 1.53 |
| 66.90 | 111.28 | 111.00 | 108.94 | 0.00 | 71.49 | - | 1.52 |

Table 8. Percentage changes in mean pressure and flow values of the **anomalous** **model** under **exercise** conditions, corresponding to maximum and minimum variations of **LCA** distal resistance ($\Delta\bar{R}_{LCA}$) relative to the baseline condition.

| LCA | $\Delta\bar{p}_{LV}\left( \% \right)$ | $\Delta\bar{p}_{Ao}\left( \% \right)$ | $\Delta\bar{p}_{LCA}\left( \% \right)$ | $\Delta\bar{p}_{RCA}\left( \% \right)$ | $\Delta\bar{q}_{RCA}\left( \% \right)$ |
| --- | --- | --- | --- | --- | --- |
| $\max\Delta\bar{R}_{LCA}=68.26 \%$ | $-0.50$ | $0.85$ | $5.60$ | $-0.52$ | $7.53$ |
| $\min\Delta\bar{R}_{LCA}=-20.49 \%$ | $-0.02$ | $-0.13$ | $-2.04$ | $-0.04$ | $8.09$ |

## 2.2 RCA resistance variation

Table 9. Time-averaged pressures ($\bar{p}_{LCA}, \bar{p}_{RCA}, \bar{p}_{LV}$, and $\bar{p}_{Ao}$, in $mmHg$), coronary flow rates ($\bar{q}_{LCA}$ and $\bar{q}_{RCA}$, in $ml/min$), and resistance values ($\bar{R}_{LCA}$ and $\bar{R}_{RCA}$, in $mmHg\times min/ml$) during **RCA** resistance variation in the **normal model** under **rest** conditions.

| ${\bar{\boldsymbol{p}}}_{\boldsymbol{LV}}$ | ${\bar{\boldsymbol{p}}}_{\boldsymbol{Ao}}$ | ${\bar{\boldsymbol{p}}}_{\boldsymbol{LCA}}$ | ${\bar{\boldsymbol{p}}}_{\boldsymbol{RCA}}$ | ${\bar{\boldsymbol{q}}}_{\boldsymbol{LCA}}$ | ${\bar{\boldsymbol{q}}}_{\boldsymbol{RCA}}$ | ${\bar{\boldsymbol{R}}}_{\boldsymbol{RCA}}$ | ${\bar{\boldsymbol{R}}}_{\boldsymbol{LCA}}$ |
| --- | --- | --- | --- | --- | --- | --- | --- |
| 59.40 | 94.15 | 92.72 | 68.58 | 41.38 | 175.17 | 0.39 | 2.24 |
| 60.70 | 96.62 | 95.16 | 92.76 | 43.15 | 83.24 | 1.11 | 2.21 |
| 61.17 | 97.57 | 96.08 | 95.09 | 43.46 | 54.60 | 1.74 | 2.21 |
| 61.26 | 98.43 | 96.96 | 96.50 | 43.79 | 40.53 | 2.38 | 2.21 |
| 61.54 | 98.51 | 97.08 | 97.33 | 43.43 | 30.76 | 3.16 | 2.24 |
| 61.64 | 99.04 | 97.56 | 98.38 | 43.60 | 13.62 | 7.23 | 2.24 |
| 61.81 | 99.45 | 97.77 | 99.43 | 43.63 | 0.00 | - | 2.24 |

Table 10. Percentage changes in mean pressure and flow values of the **normal** **model** under **rest** conditions, corresponding to maximum and minimum variations of **RCA** distal resistance ($\Delta\bar{R}_{RCA}$) relative to the baseline condition.

| RCA | $\Delta\bar{p}_{LV}\left( \% \right)$ | $\Delta\bar{p}_{Ao}\left( \% \right)$ | $\Delta\bar{p}_{LCA}\left( \% \right)$ | $\Delta\bar{p}_{RCA}\left( \% \right)$ | $\Delta\bar{q}_{LCA}\left( \% \right)$ |
| --- | --- | --- | --- | --- | --- |
| $\max\Delta\bar{R}_{RCA}=68.26 \%$ | $0.44$ | $0.95$ | $0.71$ | $2.16$ | $0.47$ |
| $\min\Delta\bar{R}_{RCA}=-87.63 \%$ | $-3.48$ | $-4.43$ | $-4.48$ | $-29.54$ | $-4.73$ |

Table 11. Time-averaged pressures ($\bar{p}_{LCA}, \bar{p}_{RCA}, \bar{p}_{LV}$, and $\bar{p}_{Ao}$, in $mmHg$), coronary flow rates ($\bar{q}_{LCA}$ and $\bar{q}_{RCA}$, in $ml/min$), and resistance values ($\bar{R}_{LCA}$ and $\bar{R}_{RCA}$, in $mmHg\times min/ml$) during **RCA** resistance variation in the **anomalous model** under **rest** conditions.

| ${\bar{\boldsymbol{p}}}_{\boldsymbol{LV}}$ | ${\bar{\boldsymbol{p}}}_{\boldsymbol{Ao}}$ | ${\bar{\boldsymbol{p}}}_{\boldsymbol{LCA}}$ | ${\bar{\boldsymbol{p}}}_{\boldsymbol{RCA}}$ | ${\bar{\boldsymbol{q}}}_{\boldsymbol{LCA}}$ | ${\bar{\boldsymbol{q}}}_{\boldsymbol{RCA}}$ | ${\bar{\boldsymbol{R}}}_{\boldsymbol{RCA}}$ | ${\bar{\boldsymbol{R}}}_{\boldsymbol{LCA}}$ |
| --- | --- | --- | --- | --- | --- | --- | --- |
| 58.00 | 92.97 | 91.97 | 82.32 | 39.25 | 212.06 | 0.39 | 2.34 |
| 59.73 | 96.69 | 95.70 | 94.01 | 41.58 | 84.06 | 1.12 | 2.30 |
| 60.25 | 97.56 | 96.55 | 95.96 | 41.69 | 54.83 | 1.75 | 2.32 |
| 60.50 | 98.19 | 97.15 | 97.02 | 41.35 | 39.83 | 2.44 | 2.35 |
| 60.56 | 98.83 | 97.80 | 98.07 | 41.63 | 32.30 | 3.04 | 2.35 |
| 60.71 | 99.23 | 98.02 | 98.77 | 41.68 | 15.21 | 6.50 | 2.35 |
| 60.95 | 99.58 | 98.46 | 99.27 | 41.80 | 0.00 | - | 2.36 |

Table 12. Percentage changes in mean pressure and flow values of the **anomalous** **model** under **rest** conditions, corresponding to maximum and minimum variations of **RCA** distal resistance ($\Delta\bar{R}_{RCA}$) relative to the baseline condition.

| RCA | $\Delta\bar{p}_{LV}\left( \% \right)$ | $\Delta\bar{p}_{Ao}\left( \% \right)$ | $\Delta\bar{p}_{LCA}\left( \% \right)$ | $\Delta\bar{p}_{RCA}\left( \% \right)$ | $\Delta\bar{q}_{LCA}\left( \% \right)$ |
| --- | --- | --- | --- | --- | --- |
| $\max\Delta\bar{R}_{RCA}=114.42 \%$ | $0.65$ | $0.76$ | $0.67$ | $1.23$ | $0.41$ |
| $\min\Delta\bar{R}_{RCA}=-87.21 \%$ | $-4.22$ | $-5.93$ | $-5.96$ | $-16.06$ | $-5.71$ |

Table 13. Time-averaged pressures ($\bar{p}_{LCA}, \bar{p}_{RCA}, \bar{p}_{LV}$, and $\bar{p}_{Ao}$, in $mmHg$), coronary flow rates ($\bar{q}_{LCA}$ and $\bar{q}_{RCA}$, in $ml/min$), and resistance values ($\bar{R}_{LCA}$ and $\bar{R}_{RCA}$, in $mmHg\times min/ml$) during **RCA** resistance variation in the **normal model** under **exercise** conditions.

| ${\bar{\boldsymbol{p}}}_{\boldsymbol{LV}}$ | ${\bar{\boldsymbol{p}}}_{\boldsymbol{Ao}}$ | ${\bar{\boldsymbol{p}}}_{\boldsymbol{LCA}}$ | ${\bar{\boldsymbol{p}}}_{\boldsymbol{RCA}}$ | ${\bar{\boldsymbol{q}}}_{\boldsymbol{LCA}}$ | ${\bar{\boldsymbol{q}}}_{\boldsymbol{RCA}}$ | ${\bar{\boldsymbol{R}}}_{\boldsymbol{RCA}}$ | ${\bar{\boldsymbol{R}}}_{\boldsymbol{LCA}}$ |
| --- | --- | --- | --- | --- | --- | --- | --- |
| 65.04 | 108.89 | 103.03 | 99.53 | 93.67 | 199.14 | 0.50 | 1.10 |
| 66.07 | 110.54 | 104.70 | 106.90 | 95.57 | 108.58 | 0.98 | 1.10 |
| 66.13 | 111.09 | 105.27 | 108.65 | 96.09 | 83.32 | 1.30 | 1.10 |
| 66.34 | 111.12 | 105.18 | 109.27 | 98.95 | 67.06 | 1.63 | 1.06 |
| 66.69 | 111.55 | 105.47 | 110.47 | 98.29 | 48.11 | 2.30 | 1.07 |
| 66.87 | 112.09 | 106.15 | 111.89 | 98.84 | 17.79 | 6.29 | 1.07 |
| 67.06 | 112.25 | 106.08 | 112.37 | 99.13 | 0.00 | - | 1.07 |

Table 14. Percentage changes in mean pressure and flow values of the **normal** **model** under **exercise** conditions, corresponding to maximum and minimum variations of **RCA** distal resistance ($\Delta\bar{R}_{RCA}$) relative to the baseline condition.

| RCA | $\Delta\bar{p}_{LV}\left( \% \right)$ | $\Delta\bar{p}_{Ao}\left( \% \right)$ | $\Delta\bar{p}_{LCA}\left( \% \right)$ | $\Delta\bar{p}_{RCA}\left( \% \right)$ | $\Delta\bar{q}_{LCA}\left( \% \right)$ |
| --- | --- | --- | --- | --- | --- |
| $\max\Delta\bar{R}_{RCA}=286.64 \%$ | $1.09$ | $1.02$ | $0.85$ | $2.84$ | $0.18$ |
| $\min\Delta\bar{R}_{RCA}=-69.33 \%$ | $-1.95$ | $-2.00$ | $-2.04$ | $-8.92$ | $-5.34$ |

Table 15. Time-averaged pressures ($\bar{p}_{LCA}, \bar{p}_{RCA}, \bar{p}_{LV}$, and $\bar{p}_{Ao}$, in $mmHg$), coronary flow rates ($\bar{q}_{LCA}$ and $\bar{q}_{RCA}$, in $ml/min$), and resistance values ($\bar{R}_{LCA}$ and $\bar{R}_{RCA}$, in $mmHg\times min/ml$) during **RCA** resistance variation in the **anomalous model** under **exercise** conditions.

| ${\bar{\boldsymbol{p}}}_{\boldsymbol{LV}}$ | ${\bar{\boldsymbol{p}}}_{\boldsymbol{Ao}}$ | ${\bar{\boldsymbol{p}}}_{\boldsymbol{LCA}}$ | ${\bar{\boldsymbol{p}}}_{\boldsymbol{RCA}}$ | ${\bar{\boldsymbol{q}}}_{\boldsymbol{LCA}}$ | ${\bar{\boldsymbol{q}}}_{\boldsymbol{RCA}}$ | ${\bar{\boldsymbol{R}}}_{\boldsymbol{RCA}}$ | ${\bar{\boldsymbol{R}}}_{\boldsymbol{LCA}}$ |
| --- | --- | --- | --- | --- | --- | --- | --- |
| 65.24 | 107.31 | 102.38 | 95.95 | 100.04 | 203.75 | 0.47 | 1.02 |
| 65.94 | 108.90 | 103.85 | 105.21 | 103.33 | 110.22 | 0.95 | 1.01 |
| 66.21 | 109.52 | 104.35 | 107.14 | 103.30 | 81.60 | 1.31 | 1.01 |
| 66.19 | 109.70 | 104.68 | 107.94 | 103.64 | 67.20 | 1.61 | 1.01 |
| 66.27 | 109.76 | 104.63 | 108.60 | 105.85 | 47.68 | 2.28 | 0.99 |
| 66.53 | 110.26 | 105.10 | 109.78 | 105.86 | 19.65 | 5.59 | 0.99 |
| 66.68 | 110.55 | 105.50 | 110.33 | 106.67 | 0.00 | - | 0.99 |

Table 16. Percentage changes in mean pressure and flow values of the **anomalous** **model** under **exercise** conditions, corresponding to maximum and minimum variations of **RCA** distal resistance ($\Delta\bar{R}_{RCA}$) relative to the baseline condition.

| RCA | $\Delta\bar{p}_{LV}\left( \% \right)$ | $\Delta\bar{p}_{Ao}\left( \% \right)$ | $\Delta\bar{p}_{LCA}\left( \% \right)$ | $\Delta\bar{p}_{RCA}\left( \% \right)$ | $\Delta\bar{q}_{LCA}\left( \% \right)$ |
| --- | --- | --- | --- | --- | --- |
| $\max\Delta\bar{R}_{RCA}=248.63 \%$ | $0.75$ | $0.77$ | $0.78$ | $2.22$ | $2.92$ |
| $\min\Delta\bar{R}_{RCA}=-70.68 \%$ | $-1.43$ | $-2.18$ | $-2.20$ | $-11.10$ | $-3.47$ |

# 3 Appendix C

This appendix summarizes the time-averaged pressures ($\bar{p}_{LCA}, \bar{p}_{RCA}, \bar{p}_{LV}$, and $\bar{p}_{Ao}$), coronary flow rates ($\bar{q}_{LCA}$ and $\bar{q}_{RCA}$), and resistance values ($\bar{R}_{LCA}$ and $\bar{R}_{RCA}$) measured during tests in which the mean aortic pressure was varied.

Table 17. Time-averaged pressures ($\bar{p}_{LCA}, \bar{p}_{RCA}, \bar{p}_{LV}$, and $\bar{p}_{Ao}$, in $mmHg$), coronary flow rates ($\bar{q}_{LCA}$ and $\bar{q}_{RCA}$, in $ml/min$), and resistance values ($\bar{R}_{LCA}$ and $\bar{R}_{RCA}$, in $mmHg\times min/ml$) during mean **aortic** pressure variation in the **normal model** under **rest** conditions.

| ${\bar{\boldsymbol{p}}}_{\boldsymbol{LV}}$ | ${\bar{\boldsymbol{p}}}_{\boldsymbol{Ao}}$ | ${\bar{\boldsymbol{p}}}_{\boldsymbol{LCA}}$ | ${\bar{\boldsymbol{p}}}_{\boldsymbol{RCA}}$ | ${\bar{\boldsymbol{q}}}_{\boldsymbol{LCA}}$ | ${\bar{\boldsymbol{q}}}_{\boldsymbol{RCA}}$ | ${\bar{\boldsymbol{R}}}_{\boldsymbol{LCA}}$ | ${\bar{\boldsymbol{R}}}_{\boldsymbol{RCA}}$ |
| --- | --- | --- | --- | --- | --- | --- | --- |
| 81.50 | 145.06 | 143.26 | 144.23 | 55.96 | 39.33 | 2.56 | 3.67 |
| 72.44 | 124.19 | 122.59 | 123.53 | 50.92 | 33.37 | 2.41 | 3.70 |
| 68.96 | 115.99 | 114.47 | 115.45 | 48.26 | 30.29 | 2.37 | 3.81 |
| 63.46 | 102.45 | 101.07 | 102.02 | 44.47 | 26.55 | 2.27 | 3.84 |
| 61.39 | 98.90 | 97.69 | 98.48 | 43.25 | 26.41 | 2.26 | 3.73 |
| 59.27 | 94.06 | 92.71 | 93.54 | 42.23 | 23.34 | 2.20 | 4.01 |
| 56.60 | 89.27 | 87.96 | 88.83 | 40.52 | 21.72 | 2.17 | 4.09 |
| 53.30 | 80.69 | 79.48 | 80.26 | 37.40 | 18.54 | 2.13 | 4.33 |
| 50.77 | 76.30 | 75.19 | 75.96 | 35.57 | 16.47 | 2.11 | 4.61 |

Table 18. Time-averaged pressures ($\bar{p}_{LCA}, \bar{p}_{RCA}, \bar{p}_{LV}$, and $\bar{p}_{Ao}$, in $mmHg$), coronary flow rates ($\bar{q}_{LCA}$ and $\bar{q}_{RCA}$, in $ml/min$), and resistance values ($\bar{R}_{LCA}$ and $\bar{R}_{RCA}$, in $mmHg\times min/ml$) during mean **aortic** pressure variation in the **normal model** under **exercise** conditions.

| ${\bar{\boldsymbol{p}}}_{\boldsymbol{LV}}$ | ${\bar{\boldsymbol{p}}}_{\boldsymbol{Ao}}$ | ${\bar{\boldsymbol{p}}}_{\boldsymbol{LCA}}$ | ${\bar{\boldsymbol{p}}}_{\boldsymbol{RCA}}$ | ${\bar{\boldsymbol{q}}}_{\boldsymbol{LCA}}$ | ${\bar{\boldsymbol{q}}}_{\boldsymbol{RCA}}$ | ${\bar{\boldsymbol{R}}}_{\boldsymbol{LCA}}$ | ${\bar{\boldsymbol{R}}}_{\boldsymbol{RCA}}$ |
| --- | --- | --- | --- | --- | --- | --- | --- |
| 86.34 | 151.48 | 140.81 | 143.97 | 151.15 | 90.21 | 0.93 | 1.60 |
| 78.45 | 136.42 | 127.11 | 129.92 | 131.05 | 82.17 | 0.97 | 1.58 |
| 72.22 | 123.40 | 115.25 | 117.71 | 115.28 | 74.81 | 1.00 | 1.57 |
| 66.49 | 111.52 | 104.55 | 106.48 | 102.74 | 67.25 | 1.02 | 1.58 |
| 63.14 | 104.12 | 97.57 | 101.56 | 93.69 | 63.89 | 1.04 | 1.59 |
| 57.60 | 91.67 | 86.51 | 89.34 | 74.52 | 54.11 | 1.16 | 1.65 |
| 51.89 | 77.91 | 74.71 | 76.03 | 42.48 | 40.85 | 1.76 | 1.86 |

Table 19. Time-averaged pressures ($\bar{p}_{LCA}, \bar{p}_{RCA}, \bar{p}_{LV}$, and $\bar{p}_{Ao}$, in $mmHg$), coronary flow rates ($\bar{q}_{LCA}$ and $\bar{q}_{RCA}$, in $ml/min$), and resistance values ($\bar{R}_{LCA}$ and $\bar{R}_{RCA}$, in $mmHg\times min/ml$) during mean **aortic** pressure variation in the **anomalous model** under **rest** conditions.

| ${\bar{\boldsymbol{p}}}_{\boldsymbol{LV}}$ | ${\bar{\boldsymbol{p}}}_{\boldsymbol{Ao}}$ | ${\bar{\boldsymbol{p}}}_{\boldsymbol{LCA}}$ | ${\bar{\boldsymbol{p}}}_{\boldsymbol{RCA}}$ | ${\bar{\boldsymbol{q}}}_{\boldsymbol{LCA}}$ | ${\bar{\boldsymbol{q}}}_{\boldsymbol{RCA}}$ | ${\bar{\boldsymbol{R}}}_{\boldsymbol{LCA}}$ | ${\bar{\boldsymbol{R}}}_{\boldsymbol{RCA}}$ |
| --- | --- | --- | --- | --- | --- | --- | --- |
| 80.14 | 143.67 | 142.54 | 142.65 | 53.32 | 38.04 | 2.67 | 3.75 |
| 72.99 | 126.30 | 125.27 | 125.58 | 48.88 | 33.87 | 2.56 | 3.71 |
| 67.66 | 114.57 | 113.60 | 113.88 | 46.02 | 32.16 | 2.47 | 3.54 |
| 63.59 | 104.45 | 103.56 | 103.92 | 43.80 | 28.51 | 2.36 | 3.64 |
| 61.05 | 98.88 | 97.97 | 98.43 | 41.74 | 28.50 | 2.35 | 3.45 |
| 59.28 | 95.21 | 94.44 | 94.54 | 40.77 | 27.20 | 2.32 | 3.48 |
| 54.55 | 84.78 | 84.14 | 84.10 | 36.73 | 24.54 | 2.29 | 3.43 |
| 51.28 | 77.96 | 77.39 | 77.25 | 33.81 | 22.60 | 2.29 | 3.42 |
| 49.50 | 75.51 | 74.96 | 74.79 | 33.05 | 22.63 | 2.27 | 3.30 |

Table 20. Time-averaged pressures ($\bar{p}_{LCA}, \bar{p}_{RCA}, \bar{p}_{LV}$, and $\bar{p}_{Ao}$, in $mmHg$), coronary flow rates ($\bar{q}_{LCA}$ and $\bar{q}_{RCA}$, in $ml/min$), and resistance values ($\bar{R}_{LCA}$ and $\bar{R}_{RCA}$, in $mmHg\times min/ml$) during mean **aortic** pressure variation in the **anomalous model** under **exercise** conditions.

| ${\bar{\boldsymbol{p}}}_{\boldsymbol{LV}}$ | ${\bar{\boldsymbol{p}}}_{\boldsymbol{Ao}}$ | ${\bar{\boldsymbol{p}}}_{\boldsymbol{LCA}}$ | ${\bar{\boldsymbol{p}}}_{\boldsymbol{RCA}}$ | ${\bar{\boldsymbol{q}}}_{\boldsymbol{LCA}}$ | ${\bar{\boldsymbol{q}}}_{\boldsymbol{RCA}}$ | ${\bar{\boldsymbol{R}}}_{\boldsymbol{LCA}}$ | ${\bar{\boldsymbol{R}}}_{\boldsymbol{RCA}}$ |
| --- | --- | --- | --- | --- | --- | --- | --- |
| 86.28 | 150.84 | 143.10 | 147.56 | 152.92 | 94.02 | 0.94 | 1.57 |
| 78.52 | 133.56 | 126.98 | 130.72 | 135.94 | 85.05 | 0.93 | 1.54 |
| 71.09 | 118.73 | 113.21 | 116.18 | 117.42 | 77.02 | 0.96 | 1.51 |
| 66.40 | 109.68 | 104.77 | 107.33 | 104.36 | 70.95 | 1.00 | 1.51 |
| 61.62 | 98.38 | 94.65 | 96.39 | 87.05 | 62.42 | 1.09 | 1.54 |
| 57.84 | 90.86 | 87.72 | 89.06 | 73.73 | 56.13 | 1.19 | 1.59 |
| 49.49 | 73.13 | 71.84 | 71.78 | 30.99 | 35.83 | 2.32 | 2.00 |
